# A Preliminary Exploratory Clinical Trial of the Efficacy of Digital Therapeutics for ADHD children in Korea

**DOI:** 10.1192/j.eurpsy.2025.1441

**Published:** 2025-08-26

**Authors:** M.-S. Shin, G.-E. Nam, C. Park, S.-H. Park, B.-N. Kim, S. Lee, S. Oh, M. J. Kang, H. W. Lee, C. Jo

**Affiliations:** 1 Psychiatry, Seoul National University Hospital; 2 DH team, Dragonfly GF Co., Ltd., Seoul, Korea, Republic Of

## Abstract

**Introduction:**

Recently, CBT-based digital therapy has been developed and used for the treatment of various psychiatric disorders, including insomnia, depression, anxiety and panic disorders, and alcohol/drug addiction. In the United States, the first game-based digital therapy for ADHD has also received FDA approval and is being used for the treatment of children and adolescents with ADHD.

**Objectives:**

We conducted a randomized controlled study to examine the effectiveness of a digital therapeutic (model named ‘ADAM-101’) for children with ADHD in Korea, which was developed by Dragonfly GF Co., Ltd.”

**Methods:**

Participants are 18 children with ADHD, aged 7 to 13 years, who are visiting the Department of Child and Adolescent Psychiatry at Seoul National University Children’s Hospital in Seoul, Korea. ADHD children with an IQ of 70 or above, who are currently taking stimulants and do not have other pediatric psychiatric disorders such as depression, anxiety disorders, tic disorders, ASD, were included in the study. They were randomly assigned to either the combined treatment group (medication + digital therapy, n = 9) or the medication-only group (n = 9). The digital therapy program was conducted using a tablet PC for 25 minutes a day, 5 days a week, for 4 weeks. Before starting the study, permission was obtained from the Institutional Review Board of Seoul National University Hospital. As a primary outcome measure, the Korean version of the Continuous Performance Test (KAT) was administered individually to the ADHD children by child clinical psychologists to assess inattention, impulsivity, and processing speed, after obtaining written agreement to participate in the study. Additionally, the Korean version f the ADHD Rating Scale-5 (K-ARS-5) was administered to the parents of the ADHD children.

**Results:**

We have not yet completed the study. Currently, out of the 18 ADHD children, 8 have completed the training and both pre- and post-assessments. All training and evaluations are expected to be completed by early October, and an analysis to verify the effectiveness of the digital therapeutic will be conducted in mid-October. Since this was not a double-blind study, we observed that, based on some children’s CPT and K-ARS-5 results, children in the combined treatment group tended to show a reduction in omission and commission errors on the CPT compared to those in the medication-only group. Additionally, there was a trend towards a reduction in inattention and hyperactivity-impulsivity scores on the K-ARS-5 in the combined treatment group.

**Conclusions:**

Despite being conducted with a small sample, these results suggest the potential efficacy of the digital therapeutic (model named ‘ADAM-101’) for Korean ADHD children, indicating its potential clinical usefulness as an adjunctive treatment tool for ADHD children

**Disclosure of Interest:**

None Declared

